# The Impact of the Antigenic Composition of Chimeric Proteins on Their Immunoprotective Activity against Chronic Toxoplasmosis in Mice

**DOI:** 10.3390/vaccines7040154

**Published:** 2019-10-18

**Authors:** Justyna Gatkowska, Katarzyna Dzitko, Bartłomiej Tomasz Ferra, Lucyna Holec-Gąsior, Malwina Kawka, Bożena Dziadek

**Affiliations:** 1 Department of Immunoparasitology, Faculty of Biology and Environmental Protection, University of Lodz, 90-237 Łódź, Poland; katarzyna.dzitko@biol.uni.lodz.pl (K.D.); malwina.kawka@biol.uni.lodz.pl (M.K.); bozena.dziadek@biol.uni.lodz.pl (B.D.); 2 Department of Molecular Biotechnology and Microbiology, Faculty of Chemistry, Gdańsk University of Technology, 80-233 Gdańsk, Poland; barferra@pg.edu.pl (B.T.F.); holec@pg.edu.pl (L.H.-G.)

**Keywords:** chimeric antigen, immunogenicity, immunoprotection, *Toxoplasma gondii*, murine experimental model

## Abstract

Toxoplasmosis may pose a serious threat for individuals with weakened or undeveloped immune systems. However, to date, there is no specific immunoprophylaxis for humans. Thus, the aim of this study was to evaluate the immunogenicity of three trivalent—SAG2-GRA1-ROP1_L_ (SGR), SAG1_L_-MIC1-MAG1 (SMM), and GRA1-GRA2-GRA6 (GGG)—and two tetravalent—SAG2-GRA1-ROP1-GRA2 (SGRG) and SAG1-MIC1-MAG1-GRA2 (SMMG)—chimeric *T. gondii* proteins, as well as their protective potential against chronic toxoplasmosis in laboratory mice. All three trivalent recombinant proteins possessed immunogenic properties, as defined by specific humoral and cellular responses in vaccinated mice characterized by the synthesis of specific IgG (IgG1/IgG2a) antibodies in vivo and the release of Th1/Th2 cytokines by stimulated splenocytes in vitro. Immunization with all three recombinant proteins provided partial protection against toxoplasmosis, although the protective capacity strongly depended on the individual antigenic composition of each preparation. The antigens providing the highest (86%) and lowest (45%) protection, SGR and SMM, respectively, were supplemented with GRA2 antigen fragment, to form the tetravalent chimeric proteins SGRG and SMMG. Further study revealed that the tetravalent preparations exhibited high immunogenic potential; however, the addition of another antigen to the recombinant protein structure had distinct effects on the protection generated, compared to that of the trivalent counterparts, depending on the antigen tested.

## 1. Introduction

Despite many years of research, toxoplasmosis, like other parasitic invasions, still lacks specific immunoprophylaxis suitable for humans. The only available registered vaccine, Toxovax, which comprises live attenuated *Toxoplasma gondii* S48 strain tachyzoites, has limited use and can only reduce the incidence of abortion and neonatal mortality due to toxoplasmosis in sheep [1,2]. The vaccine is expensive, has a short shelf life, induces rather short-lived immunity lasting approximately 18 months post-administration [3,4], and does not provide complete protection against challenge with a cyst-forming strain of the parasite [5]. Furthermore, the use of live parasites excludes this preparation from clinical use due to possible reversion of the attenuated parasite to its fully virulent variant. Thus, the search for an effective vaccine continues, since this approach would be the most reliable in protecting both humans and animals from invasion. 

Although usually asymptomatic in otherwise healthy immunocompetent individuals, *T. gondii* infection can pose a serious threat to health and life in individuals with weakened (AIDS patients, transplant recipients or those receiving immunosuppressive therapy) or underdeveloped (fetuses) immune systems. Furthermore, although it is considered clinically asymptomatic, chronic toxoplasmosis may in fact be associated with development of very serious life-controlling neurologic disorders and illnesses, such as schizophrenia [6] or depression [7]. Additionally, it has been proved in animal models—in particular, in rodents (mice and rats)—that the presence of the parasite within the central nervous system results in highly specific changes in the behavior of the intermediate host that are believed to increase the likelihood of the parasite’s transmission to the definitive hosts, which are felids. This transmission, in turn, may promote *T. gondii* spread in the environment. Thus, infected mice and rats become attracted to the smell of cat predators, while their cognitive and social behaviors remain unchanged [8,9]. Alarmingly, the influence of *T. gondii* invasion on human behavior, which is characterized by certain personality traits, has also been shown [10]. It should also be noted that toxoplasmosis in livestock not only constitutes a source of infection for humans due to the consumption of underprepared meat products but also causes great economic loses [11,12].

Due to the facts presented, the development of an effective and universal vaccine against *T. gondii* infection still remains an important task and many different approaches, such as those utilizing irradiated parasites, recombinant proteins typical for different parasite stages, or DNA vaccines [3,4], have been employed to solve the problem of specific anti-*Toxoplasma* immunoprophylaxis. However, none of these trials have led to a licensed vaccine for use in humans or/and animals to date.

One of the most recent approaches to both diagnosis and immunoprophylaxis of toxoplasmosis focuses on chimeric antigens comprising carefully selected immunodominant antigenic fragments of the parasite’s proteins. Similar to subunit vaccines composed of individual recombinant antigens, this solution circumvents the use of whole cell preparations, which may cause adverse reactions in vaccinated individuals. However, chimeric proteins have several advantages over subunit vaccines comprising individual proteins. The chimeric antigen, regardless of its length and antigenic composition, is obtained as a single product with the purity of any single-antigen recombinant protein produced in the same expression system. Considering that most subunit vaccines tested consist of several separate recombinant proteins mixed together, the amount of the producer’s proteins increases with each added antigen, which may affect immunized laboratory animals. Provided that the chimeric antigen is administered at a dose of a single antigenic ingredient of the subunit vaccine, the amount of contaminating proteins is significantly lower, which is beneficial for the vaccinated animal due to reduced risk of adverse reactions. Furthermore, it is possible to obtain a single protein containing various immunodominant fragments of parasite proteins, and production and purification of one protein is much more cost-effective [13]. 

Following one of the most modern strategies for constructing subunit vaccines, the aim of this study was to determine the immunogenic and immunoprotective efficacy of three trivalent—SAG2-GRA1-ROP1_L_ (SGR), SAG1_L_-MIC1-MAG1 (SMM), and GRA1-GRA2-GRA6 (GGG)—and two tetravalent—SAG2-GRA1-ROP1-GRA2 (SGRG) and SAG1-MIC1-MAG1-GRA2 (SMMG)—chimeric proteins and to assess the impact of their antigenic composition on the generated protection rates. The trivalent antigens used for study were also tested for their possible diagnostic utility [13,14]. All proposed experimental vaccines contained *T. gondii* proteins that are important for parasite invasion and intracellular survival and are present on the surface of the protozoon (SAG proteins), are released during invasion from specialized organelles, such as micronemes (MIC proteins), rhoptries (ROP proteins) and dense granules (GRA proteins), or are present in the parasite cyst (MAG1 protein), as previously described in detail [13,14]. Thus, the study was carried out in two steps. In the first step, the following trivalent recombinant proteins were tested: SAG2-GRA1-ROP1_L_, SAG1_L_-MIC1-MAG1 and GRA1-GRA2-GRA6. This step showed how the type and length of antigenic fragments influenced the overall performance of the resulting chimeric protein in immunological assessments and in protection against chronic parasite invasion. In the next step, the antigenic compositions conferring the highest and lowest protection against cyst formation in mice were enriched with another antigenic fragment of GRA2 to form SAG2-GRA1-ROP1-GRA2 and SAG1-MIC1-MAG1-GRA2, respectively. The resulting proteins were also tested for their immunogenic and protective capacity, to show how the addition of an extra protein impacts the tested immune parameters.

The results of the study highlight the impact of the antigenic composition of the chimeric proteins on the generated immunity and protection against *T. gondii* invasion in regard to the development of an efficient anti-*Toxoplasma* vaccine.

## 2. Materials and Methods

### 2.1. Mice

Male C3H/HeOuJ mice, 8–12 weeks of age (originating from Charles River Laboratories), were bred under conventional conditions in the animal facility of the Faculty of Biology and Environmental Protection, University of Lodz. All experimental procedures on mice were conducted according to guidelines provided and approved by the Polish Local Ethics Commission for Experiments on Animals No. 9 in Lodz (Agreements 36/ŁB718/2014 and 33/ŁB747/2015), which operates on the basis of the act issued by the Polish Ministry of Science and Higher Education. 

### 2.2. Parasites

All parasite strains were maintained according to protocols described previously. The low-virulence *T. gondii* DX strain was maintained in vivo [15] and cysts for mice challenge were obtained from the brain of chronically infected C3H/HeOuJ mouse. The highly virulent RH strain (ATCC^®^ 50174™), used to obtain native parasite antigens (*Toxoplasma gondii* lysate antigen; TLA), was maintained in vitro in Hs27 human foreskin fibroblasts (ATCC^®^ CRL-1634™) [16]. The RH strain also served as a source of DNA template for cloning *T. gondii* gene fragments for resulting chimeric proteins. 

### 2.3. Production of Chimeric Recombinant Proteins

The trivalent *T. gondii* recombinant chimeric proteins SAG2-GRA1-ROP1_L_ (SGR), SAG1_L_-MIC1-MAG1 (SMM) and GRA1-GRA2-GRA6 (GGG) were produced and purified according to procedures described previously [13,14].

The tetravalent proteins SAG2-GRA1-ROP1-GRA2 (SGRG) and SAG1-MIC1-MAG1-GRA2 (SMMG) were obtained by addition of the GRA2 antigen fragment to the compositions of the SAG2-GRA1-ROP1_L_ and SAG1_L_-MIC1-MAG1 antigens, respectively. DNA encoding the GRA2 antigen fragment was amplified from the pUET1/GRA2 [17] recombinant plasmid according to a standard PCR amplification protocol with the Phusion High-Fidelity DNA Polymerase (Thermo Fisher Scientific, Waltham, USA) using the primers SMMGFor and SMMGRev, and SGRGFor and SGRGRev (Genomed, Warsaw, Poland) (Table 1). The PCR products were inserted into the *Hind*III and *Xho*I sites of pET30/SAG1-MIC1-MAG1 or into the *Eco*RV and *Sac*I sites of pET30/SAG2-GRA1-ROP1 [13,14] using In-Fusion^®^ HD Cloning Kit (Takara Bio USA, Inc., Mountain View, USA). The resulting recombinant plasmids, pET30/SAG1-MIC1-MAG1-GRA2 (containing amino acid residues 49-311 of SAG1, amino acid residues 25-182 of MIC1, amino acid residues 30-202 of MAG1, and amino acid residues 51-185 of GRA2) and pET30/SAG2-GRA1-ROP1-GRA2 (containing amino acid residues 31-170 of SAG2, amino acid residues 26-190 of GRA1, amino acid residues 85-396 of ROP1, and amino acid residues 51-185 of GRA2) were embedded in frame between the His6-tag domains for the purification of the recombinant proteins by metal affinity chromatography.

Information regarding oligonucleotide primers used for the construction of all recombinant plasmids encoding chimeric antigens, GenBank Accession numbers of selected *T. gondii* genes and characteristics of constricted plasmids and resulting recombinant proteins is presented in the Supplementary Materials (Tables S1 and S2) [13,14,18].

*E. coli* strain Rosetta(DE3)pLacI (Merck KGaA, Darmstadt, Germany) was transformed with pET30/SAG1-MIC1-MAG1-GRA2 and pET30/SAG2-GRA1-ROP1-GRA2 recombinant plasmids and grown in TB medium supplemented with 20 µg/mL of kanamycin (Merck KGaA, Darmstadt, Germany) and 34 µg/mL of chloramphenicol (Merck KGaA, Darmstadt, Germany) overnight at 23 °C. Next, 1000 mL of TB medium, supplemented with the same antibiotics, was inoculated with 20 mL of the overnight culture. The cultures were grown with vigorous shaking at 23 °C to the optical density of 0.4 at 600 nm (OD_600_). The production of 2 chimeric proteins was then induced with isopropyl-β-D-thiogalactopyranoside (IPTG) (Merck KGaA, Darmstadt, Germany) added to a final concentration of 1 mM, and the bacteria were incubated with vigorous shaking for 18 h at the same temperature. The cells were then harvested by centrifugation. For the single purification step, pellets from 100 mL of bacterial culture were resuspended in 20 mL of buffer MA (5 M urea, 20 mM Tris, 500 mM NaCl, 5 mM imidazole, 0.1% Triton X-100, 1 mM PMSF, 2 mM MgCl2, 10 U/mL DNase, pH 7.9) (Merck KGaA, Darmstadt, Germany). The bacterial cells were disrupted by sonication, the insoluble debris was removed by centrifugation, and the protein was purified from the supernatant with a Ni^2+^-iminodiacetic acid-Sepharose column in accordance with the manufacturer’s instructions (Novagen^®^, Merck KGaA, Darmstadt, Germany).

The recombinant proteins were analyzed by standard SDS-PAGE using 10% acrylamide gels and Coomassie blue (Merck KGaA, Darmstadt, Germany) as a dye, as well as by Western blot with anti-His antibodies (Merck KGaA, Darmstadt, Germany). The concentrations of the purified proteins were determined by the Bradford method (Bradford Reagent) (Merck KGaA, Darmstadt, Germany), according to manufacturer’s manual, using bovine serum albumin as the standard. 

### 2.4. Mouse Immunization and Challenge

**Figure 1:**
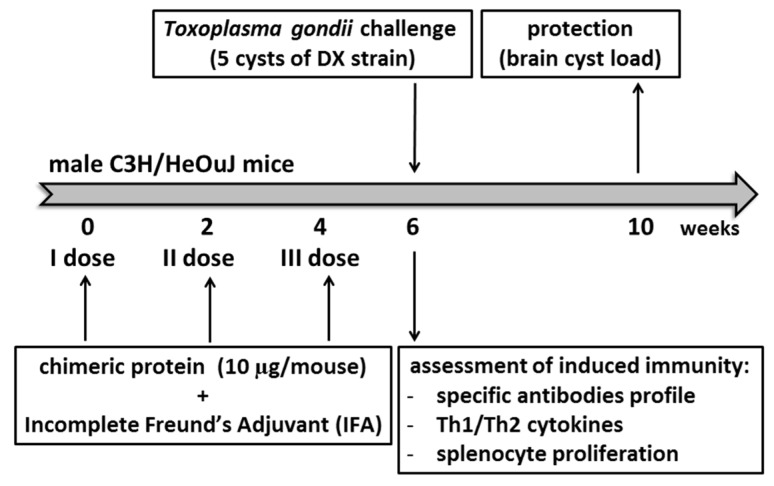
The experimental schedule of mouse immunization and challenge.

Mice were immunized subcutaneously and challenged according to a previously developed and optimized procedure [19] involving three doses of tested chimeric proteins emulsified in incomplete Freund’s adjuvant (IFA) (Merck KGaA, Darmstadt, Germany), as summarized in the Figure 1. Mice were randomly divided into experimental groups comprising 4–6 animals for immunological assessments and 9–11 animals for challenge with *T. gondii* DX cysts. Mice comprising one experimental group were injected with one tested recombinant protein administered at a dose of 10 µg. Subsequent immunological assays or parasite challenges were performed two weeks after the last antigen injection. The animals receiving only adjuvant with phosphate-buffered saline (PBS) served as a negative control. 

### 2.5. Brain Cyst Enumeration

To determine the cyst burden in control and immunized *T. gondii*-challenged mice, the animals were sacrificed one month after infection and their brains were aseptically removed and mechanically homogenized in a final volume of 2.5 mL of PBS (Merck KGaA, Darmstadt, Germany). Duplicate 25 µL samples of the resulting homogenates were microscopically evaluated for the number of cysts, and the number was calculated for the total volume of each homogenate corresponding to the individual whole mouse brain. 

### 2.6. Assessment of the IgG Response and Isotype Profile after Immunization

The humoral response of mice after immunization was characterized by the reactivity of IgG antibodies from diluted sera (1:100) with native *Toxoplasma gondii* antigen (TLA) and by the levels of chimeric antigen-specific IgG1 and IgG2a antibodies (in sera diluted from 1:100 to 1:204,800). The immunoenzymatic reactions were developed with goat anti-mouse IgG (Jackson ImmunoResearch Laboratories, Inc., West Grove, USA), goat anti-mouse IgG1 (Bio-Rad AbD Serotec GmbH, Puchheim, Germany) or goat anti-mouse IgG2a (Bio-Rad AbD Serotec GmbH, Puchheim, Germany) secondary antibodies conjugated with HRP, as described previously [19]. 

### 2.7. In Vitro Splenocyte Proliferation

Two weeks after the last immunization, the splenocytes were obtained from both control and vaccinated mice, and their proliferative response to native *T. gondii* antigen (TLA, at a final concentration of 10 µg/mL) was measured, as described previously [19]. Medium alone and concanavalin A (Merck KGaA, Darmstadt, Germany) were used as negative and positive controls of proliferation, respectively. The MTT (Merck KGaA, Darmstadt, Germany) reduction assay was used to assess the proliferative response of stimulated cells in vitro. 

### 2.8. Determination of Cytokine Production by Stimulated Splenocytes

The concentrations of cytokines in the supernatants of splenocytes from immunized and control mice were measured using commercially available OptEIA™ ELISA sets (BD Biosciences, San Jose, USA), according to manufacturer’s instructions.

### 2.9. Statistical Analysis

The obtained results, except for IgG isotype determination, are presented as the mean values for each experimental group ± standard deviation. For the IgG isotypes, the results are presented for each tested animal as the serum dilution generating an OD exceeding 0.3 [20]. All statistical analyses were performed with Statistica, ver. 12.0 (StatSoft Polska, Cracow, Poland) using the Mann-Whitney U test, and the results were considered to be statistically significant at *p* < 0.05.

## 3. Results

### 3.1. Recombinant Chimeric Proteins

The expression efficacies and calculated molecular masses of the trivalent *T. gondii* recombinant chimeric proteins SAG2-GRA1-ROP1_L_ (SGR), SAG1_L_-MIC1-MAG1 (SMM) and GRA1-GRA2-GRA6 (GGG) were described previously [13,14].

**Figure 2:**
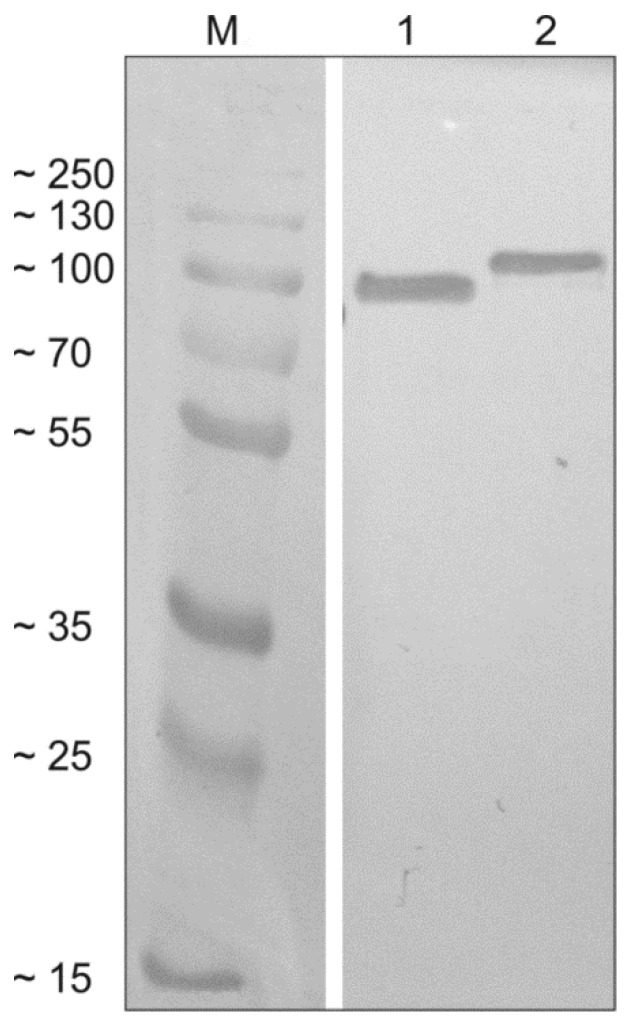
Western blot analysis of the tetravalent recombinant chimeric proteins. Recombinant proteins SAG1-MIC1-MAG1-GRA2 (SMMG; lane 1) and SAG2-GRA1-ROP1-GRA2 (SGRG; lane 2) were detected using specific anti-His antibodies and compared to the protein marker (M).

SAG1-MIC1-MAG1-GRA2 (SMMG) and SAG2-GRA1-ROP1-GRA2 (SGRG) were expressed as insoluble proteins with calculated molecular masses of 83.61 and 86.32 kDa, respectively. The procedures used yielded approximately 24 mg of SAG1-MIC1-MAG1-GRA2 protein and 15 mg of SAG2-GRA1-ROP1-GRA2 protein from a liter of induced *E. coli* culture with high purity, as confirmed by SDS-PAGE and subsequent Western blot analysis (Figure 2).

### 3.2. Protection Against Cyst Formation in Mice

**Figure 3:**
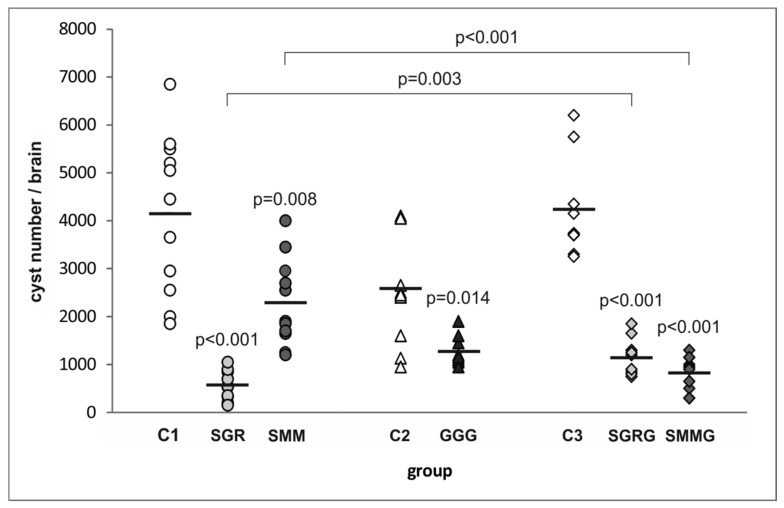
Cyst burden in the immunized and control mice challenged with *T. gondii*. *p*-values for statistically significant differences in brain cyst load of mice immunized with chimeric *T. gondii* recombinant antigens compared to their respective controls (C1, C2, C3), non-immunized mice, are given above the dots; statistically significant differences between groups immunized with trivalent antigens and their tetravalent counterparts are marked with brackets; bars represent the mean cyst numbers in experimental groups.

As shown in Figure 3, immunization with the first three tested trivalent chimeric proteins led to a significant decrease in number of brain cysts formed after *T. gondii* challenge compared to infected but non-vaccinated control mice (C1, C2). The SGR antigen proved the most potent in preventing the development of tissue cysts conferring a protection of 86% (574 cysts/brain). The other two antigens, SMM and GGG, proved to be much less efficient, since their administration resulted in a 45% (2291 cysts/brain) and 51% (1270 cysts/brain) reduction in the cyst burden, respectively, compared to their respective unvaccinated controls (C1—4150 cysts/brain, C2—2588 cysts/brain). 

The two antigens providing the highest and lowest protection rates—SAG2-GRA1-ROP1_L_ (SGR) and SAG1_L_-MIC1-MAG1 (SMM), respectively—were further tested as tetravalent proteins with the same GRA2 antigen fragment added to their C-terminal ends (SGRG and SMMG, respectively). The vaccination of mice with SMMG resulted in an 81% (822 cysts/brain) decrease in cyst burden compared to control unvaccinated animals (C3—4237 cysts/brain). Thus, the addition of a fourth antigenic immunodominant fragment to SMM led to a drastic, statistically significant increase (by 36%) in the protective capacity of the resulting SMMG protein compared to its trivalent counterpart. Interestingly, SGRG antigen administration led to a 73% (1145 cysts/brain) reduction in cyst load compared to controls (C3), which means that the tetravalent antigen provided protection that was 13% lower than its trivalent counterpart (SGR). Furthermore, the difference in protective capacity between SGR and SGRG antigens was statistically significant.

### 3.3. Analysis of the IgG Production and Isotype Profile

**Figure 4:**
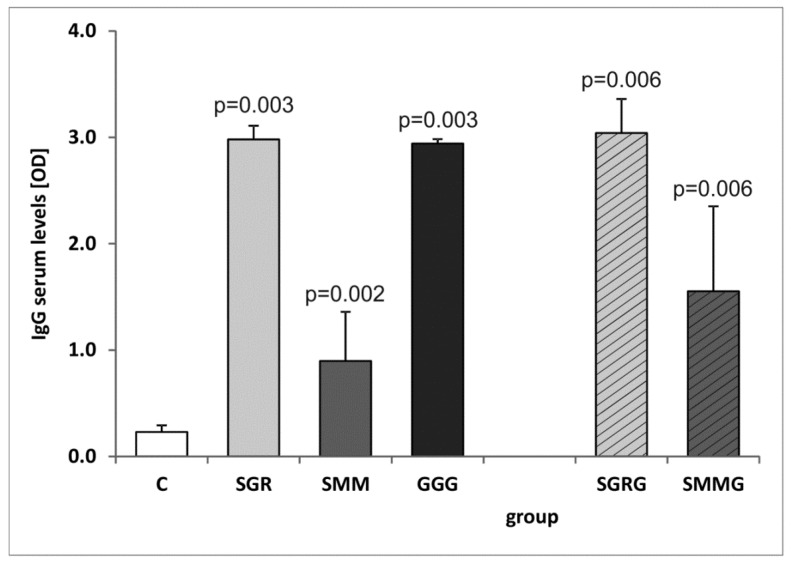
Reactivity of specific IgG antibodies from murine control and immune sera with *Toxoplasma gondii* antigen (TLA). *p*-values for statistically significant differences in reactivity of sera from immunized mice compared to control non-immunized group (C) are given above bars.

The assessment of the whole serum IgG reactivity revealed that each tested immunized mouse, regardless of the chimeric antigen used for vaccination, developed IgG immunoglobulins capable of recognizing their native counterparts present in the TLA (Figure 4). Notably, the reactivity of the SMM protein was significantly lower compared to the other two trivalent antigens (*p* = 0.004). The addition of another antigenic component to the protein structures did not significantly increase their reactivity compared to their trivalent base. Again, the use of SMMG as a detection antigen resulted in significantly lower OD values compared to SGRG (*p* = 0.029).

Further analysis of the IgG isotypes produced (Table 2) revealed that all immunized animals produced recombinant-antigen-specific IgG1 and IgG2a antibodies. The determined titer values differed greatly between individuals, even within one experimental group. The comparison of the IgG subclasses showed the statistically significant predominance of the IgG1 isotype in the sera from mice of each experimental group immunized with the tested recombinant antigens (*p* = 0.032 for SGR, *p* = 0.026 for SMM, *p* = 0.008 for GGG, *p* = 0.029 for SGRG and *p* = 0.029 for SMMG), although the calculated indexes of IgG1/IgG2a showed high variance within and between groups. The most variable results were obtained with the sera from SMM vaccinated animals, with one mouse producing equal amounts of IgG1 and IgG2a antibodies. A comparison of sera reactivity in regard to each IgG isotype revealed no statistically significant differences between the experimental groups (*p* > 0.1 for both the IgG1 and IgG2a immunoglobulin isotypes). None of the control animals receiving PBS/adjuvant produced IgG1 or IgG2a antibodies against any recombinant antigen used for immunization (reactivity <1:100).

### 3.4. Cellular Response Induced by Vaccination

Analysis of in vitro proliferation of splenocytes from immunized and control mice stimulated with TLA (Table 3) revealed that the mean proliferative response of cells from vaccinated mice was significantly higher than the response of the spleen cells from control mice for SMM and GGG chimeric antigens (*p* = 0.038 and *p* = 0.003, respectively). Splenocytes from mice vaccinated with the SGR protein showed no proliferative response to TLA compared to the TLA-stimulated control. Even the addition of the GRA2 antigen fragment to the protein structure (SGRG) did not improve the reactivity. Surprisingly, after the addition of the GRA2 fragment encoded by exon 2 to the SMM recombinant antigen (SMMG), the proliferative response of splenocytes from vaccinated mice was lower compared to that of the trivalent preparation and was not statistically significant. For all of the mouse groups tested, except for the group immunized with SGR (*p* = 0.053), the proliferation values from cultured cells stimulated with TLA were significantly higher compared to those of the splenocytes cultured in medium alone (*p* = 0.009 for SMM and GGG, *p* < 0.001 for SGRG and *p* = 0.017 for SMMG). Notably, there was no statistically significant difference in proliferative response of the splenocytes from non-immunized mice cultured in the presence of TLA and medium alone (*p* = 0.341).

### 3.5. Determination of IL-2, IFN-γ and IL-10 in Vitro Synthesis

**Figure 5:**
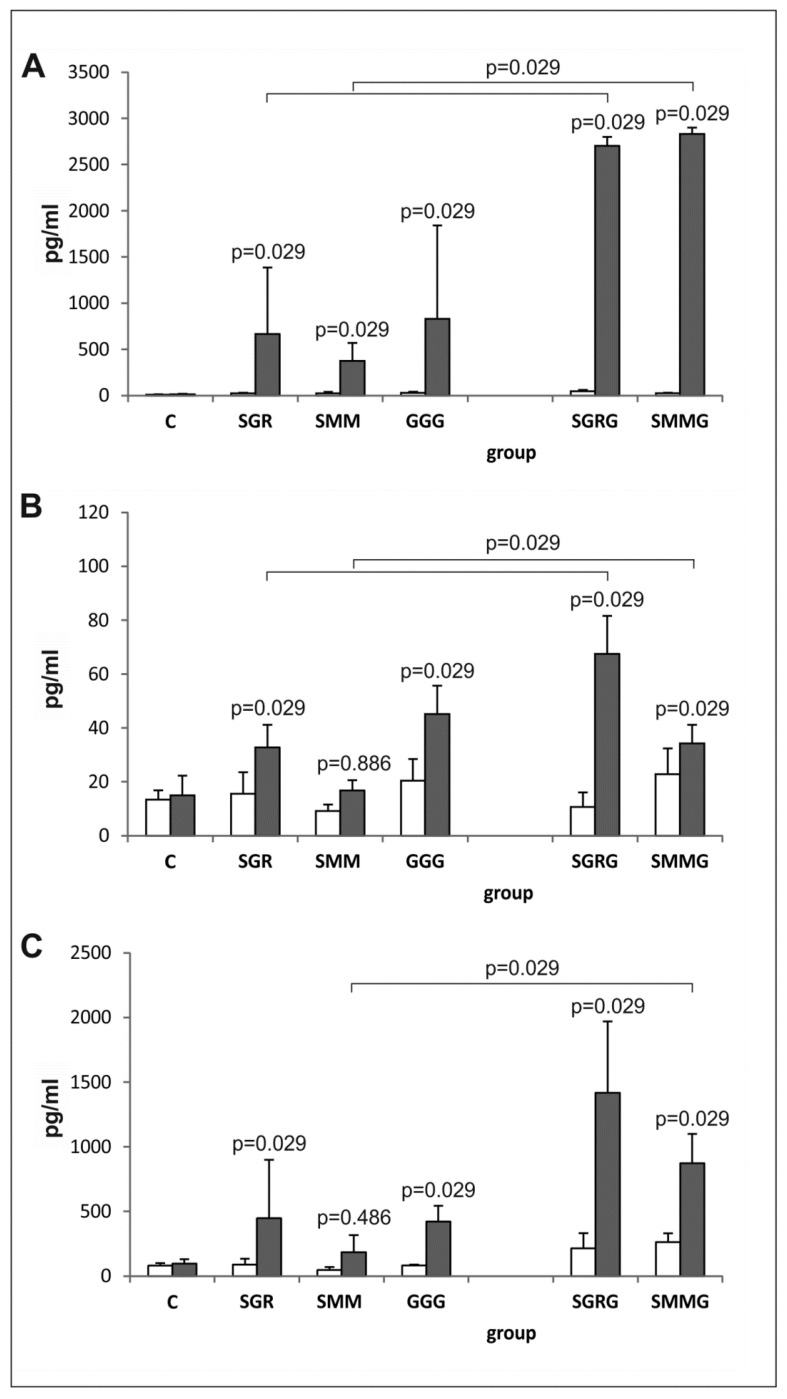
In vitro cytokine synthesis by splenocytes from immunized and control mice. (**A**) IFN-γ, (**B**) IL-2 and (**C**) IL-10 in vitro synthesis by splenocytes from mice immunized with chimeric *T. gondii* antigens and control mice in unstimulated (□) and TLA-stimulated (■) cultures; p values for statistically significant differences in cytokine production after TLA stimulation compared to control splenocytes from non-immunized mice (**C**) are given above bars; statistically significant differences in cytokine levels between groups immunized with trivalent antigens and their tetravalent counterparts after TLA stimulation are marked with brackets.

The results obtained revealed that all of the antigens tested induced a strong and specific immune response in vaccinated mice, manifesting in the in vitro synthesis of cytokines typical for the Th1 (IFN-γ, IL-2) and Th2 (IL-10) types of immunity from splenocytes stimulated with native *Toxoplasma* antigens (TLA). Despite intergroup variations, the mean concentrations of the cytokines in the supernatants of stimulated splenocytes from immunized mice were statistically higher than the concentrations measured in the supernatants collected from the spleen cell cultures of control mice, except for IL-2 and IL-10 release by lymphocytes from SMM-vaccinated mice (Figure 5).

Moreover, the production of cytokines by the splenocytes from the immunized mice in response to antigenic stimulation was statistically higher compared to that in the presence of medium alone (*p* = 0.029 for all antigens) with the exception of IL-2 in SMMG-vaccinated mice (*p* = 0.114) and IL-10 in SGR-vaccinated mice (*p* = 0.057). 

Notably, the pattern of cytokine release in all mice immunized with trivalent antigens was similar, regardless of the analyzed cytokine. Although the obtained results suggested lower cytokine production by the TLA-stimulated splenocytes from the SMM-vaccinated mice compared to those from the SGR- and GGG-immunized animals, the differences were only statistically significant for IL-2 (*p* = 0.029). 

The splenocytes obtained from mice immunized with tetravalent chimeric proteins containing the GRA2 antigenic fragment (SGRG and SMMG) released more cytokines after TLA stimulation compared to the splenocytes obtained from mice immunized with their trivalent counterparts; however, IL-10 was an exception to this in SGRG-vaccinated mice (*p* = 0.057).

## 4. Discussion

The aim of the study was to evaluate the immunogenic and immunoprotective capacity of several chimeric recombinant proteins of *T. gondii* as candidates for an effective vaccine against toxoplasmosis. Considering the possible serious consequences of parasitosis in individuals with weakened (e.g., immunocompromised) or undeveloped (fetuses) immune systems and the shortcomings of the currently used anti-parasitic drug therapy that is unable to prevent invasion or eliminate tissue cysts from infected hosts, immunoprophylaxis should be the most effective method to counter *T. gondii* infection. A universal vaccine should protect hosts, including humans, from acute invasion and congenital toxoplasmosis. Thus, the vaccine should reduce the incidence of infection in both farm animals meant for human consumption and cats responsible for oocyst shedding and environmental contamination with infectious forms of *T. gondii*, which, in turn, is expected to minimize parasite transmission to all hosts, including humans [21]. However, despite many years of research, the construction of an effective and universal anti-*Toxoplasma* vaccine for use in both humans and animals remains an elusive goal. The main obstacle is the selection of appropriate antigenic composition that is capable of inducing strong protective immunity and a delivery system (the route of immunization, the choice of adjuvant) to ensure its efficient presentation to the immune system. 

The most effective approach to vaccine development to date involves live attenuated parasites (Toxovax); however, this approach is still inadequate and cannot be used in humans due to safety concerns. Likewise, although the whole parasite lysate (TLA) or excreted/secreted parasite antigens generate protective immunity that results in high protection rates [22,23,24,25], the possibility of adverse reactions in the vaccinated individuals due to undefined antigenic composition excludes these vaccines from further research as vaccines for humans. However, these examples illustrate that multi-antigenic composition has a much greater chance of generating efficient immune responses to protect from *T. gondii* invasion. Furthermore, recombinant proteins administered concurrently can exhibit synergistic effects in eliciting protective immune responses [26].

Recombinant chimeric proteins offer a well-defined, reproducible and relatively inexpensive tool for vaccine studies compared to continuous parasite cultures. However, the main issues with the use of recombinant chimeric proteins are the selection of appropriate single antigens and their length, which may influence their immunogenicity and generated protection. Thus, this study focused on determining the immunogenic and immunoprotective activities of the tested preparations and on assessing the impact of their antigenic composition in terms of the single antigens used and their lengths. The study was partially based on our previous experiments on the diagnostic value of the three trivalent antigens tested (SGR, SMM, GGG) [13,14]. The basic principle behind the use of recombinant proteins as vaccines is that they induce specific immunity that is effective against the native antigens present in the actual infective agent. By analogy, the recombinant antigenic equivalent should be recognized and react with the products of immunity triggered by the native antigen. Thus, one of the most important features of the recombinant proteins used for immunoprophylaxis, aside from high immunogenicity, is their close similarity to their native counterparts. Furthermore, the highly immunogenic recombinant antigens have proven to be useful as potential diagnostic tools, for example, SAG1 used in commercially available tests (e.g., ADVIA Centaur *Toxoplasma* G assay), since they constitute a large portion of parasites antigens and/or they are involved in parasite invasion and intracellular survival [27]. Thus, the three vaccine candidates were thoroughly tested for their ability to react with the specific anti-*T. gondii* antibodies present in the sera of *Toxoplasma*-seropositive individuals from different species, and the three antigens most effective in toxoplasmosis detection [13,14] were studied for their immunoprotective capacity.

All trivalent recombinant chimeric antigens tested in this study—SAG2-GRA1-ROP1_L_ (SGR), SAG1_L_-MIC1-MAG1 (SMM) and GRA1-GRA2-GRA6 (GGG)—proved immunogenic, as shown by their ability to induce production of antigen-specific antibodies in vivo and/or cytokines in vitro in antigen-stimulated splenocyte cultures. All three chimeric antigens triggered strong humoral responses characterized by the synthesis of antigen-specific IgG1 and IgG2 antibodies, which is typical for Th2 and Th1 responses, respectively, with the predominance of the IgG1 isotype, which we observed in our previous studies regardless of the type of adjuvant used [19,20]. However, the most important finding is that vaccine antigens induced immune response components capable of recognizing and reacting with the native antigens present in the TLA preparation. Although all three chimeric antigens triggered immune responses, their reactivity differed depending on the parameter tested. In regard to specific IgG antibodies and cytokines, the SMM recombinant protein exhibited the weakest reaction with native antigens. One explanation is the presence of MAG1 antigen in the chimeric structure. MAG1, although is expressed in tachyzoites, is abundant in the bradyzoite stage, but it may not be present or may only be present in trace amounts in the TLA preparation, which in turn, influences the obtained results [28]. However, this explanation is not supported by the results of the proliferation assay with the same native antigen, which revealed significant proliferative responses of splenocytes obtained from mice immunized with SMM antigen. The simplest explanation may be the extended exposure to native antigen stimulation. The most conclusive results in regard to the vaccine capacity of tested antigens were obtained in the in vivo protection experiments. All three antigens provided partial protection against chronic toxoplasmosis in mice, but their efficiencies differed. The SMM composition proved the least potent in preventing tissue cyst formation, although SAG1 and MAG1 recombinant proteins of similar length, and accompanied by GRA1 in a mixture, decreased tissue cyst formation in mice by 89% in previous studies [22]. Thus, the most likely explanation is that the exchange of one antigen and its length significantly influenced the resulting protective activity of the chimeric protein, although the MIC1 antigen appeared highly promising due to the results obtained by other research teams [29,30]. It is also important to remember that, to a certain degree, chimeric antigens represent an artificial product constructed experimentally, which may influence their folding and conformational stability and in turn impact immunogenicity and immune polarization [31]. These results emphasize the importance of each antigenic component used and its length on the overall activity of the resulting fusion proteins, even if each antigen is individually highly immunogenic, such as the SAG1, MIC1 and MAG1 antigens [29,30,32,33,34].

The GRA1-GRA2-GRA6 [GGG] recombinant protein proved only slightly more efficient compared to SMM, despite its ability to trigger a strong immune response in immunized mice. Even strong immune responses against three GRA antigens, which are important for the invasion process, do not result in satisfactory protection against toxoplasmosis in mice. It is likely that other GRA antigens can compensate for the functions blocked by the specific components of the immune system induced by vaccination, which is a phenomenon observed previously in regard to ROP2 and ROP4 antigens [18]. SGR, the most potent vaccine candidate, contained relatively long antigenic fragments, especially ROP1, which is considered a good vaccine candidate [35], and it proved capable of inducing both strong immune responses and protection rates. Intriguingly, the results obtained overlap with the tests on the diagnostic utility of fusion proteins, in which higher mean and average absorbance values were usually noted for the chimeric antigens containing longer fragments of immunodominant regions of *T. gondii* proteins [13]. Interestingly, DNA immunization of BALB/c mice with a homogeneous mixture of plasmids encoding short microneme antigens fragments resulted in strong protective immunity leading to an 84% reduction of cyst burden after *T. gondii* challenge [36]. These conclusions together suggest that the length of the antigenic components used combined with the antigen type and delivery system play an important role for both the antigenicity and immunogenicity of vaccine preparation.

Since the aim of the study was also to determine the importance of the number of antigenic components in the final structure, the GRA2 portion was added to chimeric antigens with the strongest and the weakest immunoprotective capacity. According to previous works, this antigen can induce strong protective immunity, leading to significant protection against chronic invasion in mice [37,38]. However, the inclusion of an extra antigenic fragment had a distinct impact on the immunogenicity and protective activity of the obtained tetravalent preparations. The addition of the GRA2 fragment did not improve the humoral response, defined by the levels of specific IgG antibodies and IgG isotypes titers, or the proliferative response of splenocytes from immunized mice. However, splenocytes from vaccinated mice released significantly more cytokines, especially IFN-γ and IL-2, in response to TLA stimulation. Notably, IFN-γ and IL-2, which are typically evaluated in vaccine studies as indicators of the Th1 response, are fundamental for protection against *T. gondii* invasion [39]. On the other hand, IL-10, which is often assessed as a marker of Th2 response, in principle plays an important regulatory role during parasite infection [40,41]. Intriguingly, greater in vitro production of IFN-γ did not necessarily correlate with higher protection conferred in vivo. Although the addition of the fourth immunogenic fragment to the SMM structure significantly improved its immunoprotective capacity, it actually significantly reduced the protection rates provided by the SGRG chimeric antigen compared to the trivalent version (SGR). It has been observed previously [42] that although immunization of mice with two chimeric antigens resulting from separately administered plasmids provided high protection rates in vaccinated animal groups, the protective effect was completely abolished when the plasmids were administered concomitantly. These observations point out the difficulty in predicting appropriate antigenic vaccine composition. Indeed, even use of antigens considered indispensable for parasite virulence may prove inadequate to protect animals from *T. gondii* invasion [43]. Additionally, immunity generated by the tested vaccine preparation may prove efficient against only certain *T. gondii* genotypes [42].

Finally, the high protection rates obtained in the study, ranging from 73% to 86% for three out of five chimeric antigens, have been reported by other research teams testing chimeric *T. gondii* proteins as potential vaccines against toxoplasmosis. For instance, Yang et al. [44] noticed that 73% of mice immunized with rSAG1/2 chimeric protein survived at least 28 days after the lethal challenge with RH strain of *T. gondii* and the same antigen administered in poly(lactide-co-glycolide) microparticles protected 70% of mice from lethal toxoplasmosis after only a single dose [45]. However, higher protection rates were also noted for chimeric antigens resulting from DNA vaccination. Rosenberg et al. [42], reported a 93.5% reduction of brain cyst load in mice vaccinated with the DNA vaccine encoding chimeric proteins containing GRA3-GRA7-M2AP antigenic sequences from *T. gondii*. The highest protection against lethal parasite challenge, even reaching 100%, was noted by Liu et al. [46] after administration of the multi-epitope DNA vaccine encoding fragments of four *T. gondii* antigens: SAG1, GRA1, GRA4, and GRA2. These results suggest again that chimeric antigens, especially multi-antigenic preparations, may constitute a very promising immunoprophylactic tool.

## 5. Conclusions

The results of this work emphasize several important points connected to the design of an efficient anti-*T. gondii* vaccine. First, the results of the study demonstrate the importance of the antigenic composition of the prospective vaccine and show that multi-antigenic preparations consisting of long protein fragments are more potent in the protection against parasite invasion in animal models. Furthermore, the work highlights that actual performance in living organisms cannot be accurately predicted, regardless of the approach used in designing the vaccine. Moreover, the results of immune tests, such as antibody profiles and cytokine release, should only be considered to be supportive data on the immunogenicity of certain antigens or their ability to trigger specified immune responses (Th1/Th2) to be used for further study, since these assessments do not necessarily correspond to the protection observed in vivo. Finally, the described results emphasize the paramount role of in vivo protection assays, which produce the most conclusive results for supporting further anti-*T. gondii* vaccine development. 

Taken together, the recombinant chimeric proteins represent more cost-effective and promising tools compared to single recombinant antigens for further studies on toxoplasmosis immunoprophylaxis. 

## Figures and Tables

**Table 1:** Oligonucleotide primers used for the amplification of *gra2* gene fragments.

Gene Fragment	Primer Name	Primer Sequence	Corresponding Protein Residues
*gra2*	SMMG-For	5′-GGGATCTGGTAAGCTGGGAAAAGGTGAACATACACCACC-3′	GRA2 51-185 (135 aa)
	SMMG-Rev	5′-GGTGGTGGTGCTCGATCTGCGAAAAGTCTG-3′	(cloning to the pET30/SAG1-MIC1-MAG1)
	SGRG-For	5′-TGGATCGCAAGCGATGGGAAAAGGTGAACATACACCACC-3′	GRA2 51-185 (135 aa)
	SGRG-Rev	5′-CAAGCTTGTCGACGGTCTGCGAAAAGTCTGGGACGGGCA-3′	(cloning to the pET30/SAG2-GRA1-ROP1)

**Table 2:** Titers of IgG1 and IgG2a antibodies in the sera of vaccinated mice.

	Trivalent Chimeric Proteins			Tetravalent Chimeric Proteins		
	IgG1	IgG2a	IgG1/IgG2a	IgG1	IgG2a	IgG1/IgG2a
	**SGR**			**SGRG**		
mouse 1	51,200	3200	16/1	>204,800	6400	>32/1
mouse 2	>204,800	12,800	>16/1	204,800	102,400	>2/1
mouse 3	>204,800	102,400	>2/1	204,800	12,800	16/1
mouse 4	102,400	25,600	4/1	102,400	6400	16/1
mouse 5	102,400	6400	16/1	-	-	-
	**SMM**			**SMMG**		
mouse 1	>204,800	51,200	4/1	>204,800	51,200	>4/1
mouse 2	>204,800	204,800	>1/1	102,400	3200	32/1
mouse 3	102,400	12,800	8/1	>204,800	25,600	>8/1
mouse 4	>204,800	51,200	>4/1	204,800	12,800	>16/1
mouse 5	102,400	1600	64/1	-	-	-
mouse 6	204,800	6400	32/1	-	-	-
	**GGG**					
mouse 1	>204,800	51,200	>4/1	-	-	-
mouse 2	>204,800	102,400	>2/1	-	-	-
mouse 3	204,800	25,600	8/1	-	-	-
mouse 4	>204,800	12,800	>16/1	-	-	-
mouse 5	204,800	6400	>32/1	-	-	-

**Table 3:** Proliferative response of spleen cells of vaccinated and control mice stimulated with TLA in vitro.

Antigen/Group	Mean % in Proportion to TLA Stimulated Control Cells ± SD ^*^			
	Control	SGR	SMM	GGG
medium	87.32 ± 17.72	87.02 ± 16.29	96.54 ± 14.31	106.21 ± 20.66
TLA	100.00 ± 17.34	99.33 ± 15.29	121.56 ± 26.19 *	130.84 ± 19.63 *
		**SGRG**	**SMMG**	-
medium		76.22 ± 2.20	88.35 ± 20.29	-
TLA		104.25 ± 2.62	109.94 ± 14.19	-

* statistically significant differences compared to control (phosphate-buffered saline (PBS)/incomplete Freund’s adjuvant (IFA)-injected mice).

## Supplementary Materials

The following are available online at https://www.mdpi.com/2076-393X/7/4/154/s1, Table S1: oligonucleotide primers used for the construction of the recombinant plasmids encoding chimeric antigens, Table S2: GenBank Accession numbers of selected *T. gondii* genes and characteristics of constructed plasmids and resulting recombinant proteins.
