# Supplementary material for: The Impact of the Antigenic Composition of Chimeric Proteins on Their Immunoprotective Activity against Chronic Toxoplasmosis in Mice
Source: Vaccines (Basel). 2019 Oct 18;7(4):154. doi: 10.3390/vaccines7040154 (PMC6963210; doi:10.3390/vaccines7040154)
Supplement: Supplementary file 1 [file vaccines-07-00154-s001.pdf]

Table S1. Oligonucleotide primers used for the construction of the recombinant plasmids encoding chimeric antigens.

T. Gondii Gene(s)	Primer Name	Primer Sequence	Underlined Sequence	Template for Amplification
		**pET30/SAG2-GRA1-ROP1$_{L}$** [14]		
sag2	S21 (forward)	5′-GACAGCACAGATCTGACGCCAGCGCCCATTG-3′	*Bgl*II and fragment of *sag2*	pUET1/SAG2
	S22 (reverse)	5′-GTTGTCGCCGCCTTCCGTGAGAGACACAGG -3′	fragments of *gra1* and *sag2*	
gra1	G11 (forward)	5′-CCTGTGTCTCTCACGGAAGGCGGCGACAAC-3′	fragments of *sag2* and *gra1*	pUET1/GRA1
	G12 (reverse)	5′-CGGGCCTCTGACAGGCTCTCTCTCCTG-3′	fragments of *rop1* and *gra1*	
rop1l	R11 (forward)	5′-CAGGAGAGAGAGAGCCTGTCAGAGGCCCG-3′	fragments of *gra1* and *rop1*	pUET1/ROP1
	R12 (reverse)	5′-CGGCTCCGATATCGCTTGCGATCCATCATCCTG-3′	*Eco*RV and fragment of *rop1*	
sag2/gra1	S21 (forward)	same as above	same as above	
	G12 (reverse)	same as above	same as above	*pre-sag2/gra1*
sag2/gra1/rop1l	S21 (forward)	same as above	same as above	*pre-sag2/gra1/rop1l*
	R12 (reverse)	same as above	same as above	
		**pET30/SAG1$_{L}$-MIC1-MAG1** [13]		
		(PCR product was inserted into the *Bgl*II and *Bsm*I sites of the pUET1/MIC1-MAG1 [18]. Next the gene *sag1l-mic1-mag1* was sub-cloned into the pET30 Ek/LIC using *Bgl*II and *Hin*dIII.)		
sag1l	S11 (forward)	5′-CAGCCCAGATCTAGATCCCCCTCTTGTTGCC-3′	*Bgl*II and fragment of *sag1*	pUET1/SAG1
	S12 (reverse)	5′-GAATGAGAATGCGACGCAGCCGATTTTGCTGAC-3′	*Bsm*I and fragments of *mic1* and *sag1*	
		**pET30/GRA1-GRA2-GRA6** [13]		
gra1	G11 (forward)	5′-GACAGCACAGATCTGGAAGGCGGCGACAAC-3′	*Bgl*II and fragment of *gra1*	pUET1/GRA1
	G12 (reverse)	5′-GTGTATGTTCACCTTTTCCCTCTCTCTCTCCTGTTAG-3′	fragments of *gra2* and *gra1*	
gra2	G23 (forward)	5′-CTAACAGGAGAGAGAGAGAGGGAAAAGGTGAACATACACC-3′	fragments of *gra1* and *gra2*	pUET1/GRA2
	G24 (reverse)	5′-GCTGCGACACGGACCTGCGAAAAGTCTGG-3′	fragments of *gra6* and *gra2*	
gra6	G65 (forward)	5′-CCAGACTTTTCGCAGGTCCGTGTCGCAGC-3′	fragments of *gra2* and *gra6*	pUET1/GRA6
	G66 (reverse)	5′-CGGCGCCGATATCGCATAATCAAACACATTCACACG-3′	*Eco*RV and fragment of *gra6*	
gra1/gra2	G11 (forward)	same as above	same as above	
	G24 (reverse)	same as above	same as above	*pre-gra1/gra2*
gra1/gra2/gra6	G11 (forward)	same as above	same as above	*pre-gra1/gra2/gra6*
	G66 (reverse)	same as above	same as above	
		**pET30/SAG2-GRA1-ROP1$_{L}$-GRA2**		
gra2	SGRG-For	5′-TGGATCGCAAGCGATGGGAAAAGGTGAACATACACCACC-3′	fragment of *gra2*	pUET1/GRA2
	SGRG-Rev	5′-CAAGCTTGTCGACGGTCTGCGAAAAGTCTGGGACGGGCA-3′	fragment of *gra2*	
		**pET30/SAG1$_{L}$-MIC1-MAG1-GRA2**		
gra2	SMMG-For	5′-GGGATCTGGTAAGCTGGGAAAAGGTGAACATACACCACC-3′	fragment of *gra2*	pUET1/GRA2
	SMMG-Rev	5′-GGTGGTGGTGCTCGATCTGCGAAAAGTCTG-3′	fragment of *gra2*	

Table S2. GenBank Accession numbers of selected *T. gondii* genes and characteristics of constructed plasmids and resulting recombinant proteins.

Plasmid (size)	Amino acid residues	Protein characteristic	GeneBank Accession No.
pET30/SAG2-GRA1-ROP1L (7245 bp)	31-170 SAG2<br>26-190 GRA1<br>85-396 ROP1	677 aa<br>Mw 72.21 kDa<br>pI 5.18	M33572.1 (*sag2*)<br>M26007.1 (*gra1*)<br>M71274.1 (*rop1*)
pET30/SAG1L-MIC1-MAG1 (7152 bp)	49-311 SAG1<br>25-182 MIC1<br>30-202 MAG1	646 aa<br>Mw 69.21 kDa<br>pI 5.73	S76248.1 (*sag1*)<br>Z71786.1 (*mic1*)<br>U09029.1 (*mag1*)
pET30/GRA1-GRA2-GRA6 (6867 bp)	26-190 GRA1<br>51-185 GRA2<br>40-230 GRA6	551 aa<br>Mw 59.09 kDa<br>pI 5.23	M26007.1 (*gra1*)<br>M99392.1 (*gra2*)<br>L33814.1 (*gra6*)
pET30/SAG2-GRA1-ROP1L-GRA2 (7632 bp)	31-170 SAG2<br>26-190 GRA1<br>85-396 ROP1<br>51-185 GRA2	806 aa<br>Mw 86.32 kDa<br>pI 5.56	M33572.1 (*sag2*)<br>M26007.1 (*gra1*)<br>M71274.1 (*rop1*)<br>M99392.1 (*gra2*)
pET30/SAG1L-MIC1-MAG1-GRA2 (7548 bp)	49-311 SAG1<br>25-182 MIC1<br>30-202 MAG1<br>51-185 GRA2	778 aa<br>Mw 83.61 kDa<br>pI 6.02	S76248.1 (*sag1*)<br>Z71786.1 (*mic1*)<br>U09029.1 (*mag1*)<br>M99392.1 (*gra2*)
